# HIV testing within general practices in Europe: a mixed-methods systematic review

**DOI:** 10.1186/s12889-018-6107-0

**Published:** 2018-10-22

**Authors:** Jessika Deblonde, Dominique Van Beckhoven, Jasna Loos, Nicole Boffin, André Sasse, Christiana Nöstlinger, Virginie Supervie, HERMETIC Study Group, Hanne Apers, Jessika Deblonde, Anda Ķīvīte, Jasna Loos, Lise Marty, Christiana Nöstlinger, Daniela Rojas Castro, Virginie Supervie, Dominique Van Beckhoven

**Affiliations:** 1 Sciensano, Health Services Research, Juliette Wytsmanstraat 14, 1050 Brussels, Belgium; 2 Department of Public Health, Institute of Tropical Medicine, Nationalestraat 155, 2000 Antwerp, Belgium; 3 Faculty of Psychology, University of Vienna, Vienna, Austria; 4 Institut Pierre Louis d’Epidémiologie et de Santé Publique, INSERM, Sorbonne Université, 56 Bd. Vincent Auriol, CS 81393, 75646 Paris Cedex 13, France

**Keywords:** HIV, HIV testing, Primary care, General practitioner, Europe

## Abstract

**Background:**

Late diagnosis of HIV infection remains a key challenge in Europe. It is acknowledged that general practitioners (GPs) may contribute greatly to early case finding, yet there is evidence that many diagnostic opportunities are being missed. To further promote HIV testing in primary care and to increase the utility of available research, the existing evidence has been synthesised in a systematic review adhering to the PRISMA guidelines.

**Methods:**

The databases PubMed, Scopus and Embase were searched for the period 2006–2017. Two authors judged independently on the eligibility of studies. Through a mixed-methods systematic review of 29 studies, we provide a description of HIV testing in general practices in Europe, including barriers and facilitators.

**Results:**

The findings of the study show that although various approaches to target patients are used by GPs, most tests are still carried out based on the patient’s request. Several barriers obstruct HIV testing in general practice. Included are a lack of communication skills on sexual health, lack of knowledge about HIV testing recommendations and epidemic specificities, difficulties with using the complete list of clinical HIV indicator diseases and lack of experience in delivering and communicating test results. The findings also suggest that the provision of specific training, practical tools and promotion programmes has an impact on the testing performance of GPs.

**Conclusions:**

GPs could have an increased role in provider-initiated HIV-testing for early case finding. To achieve this objective, solutions to the reported barriers should be identified and testing criteria adapted to primary healthcare defined. Providing guidance and training to better identify priority groups for HIV testing, as well as information on the HIV epidemic’s characteristics, will be fundamental to increasing awareness and testing by GPs.

**Electronic supplementary material:**

The online version of this article (10.1186/s12889-018-6107-0) contains supplementary material, which is available to authorized users.

## Background

From a clinical and public health perspective, early HIV care and treatment are associated with viral suppression, improved health outcomes and reductions in transmission risks [1–3]. However, due to significant barriers to HIV testing, many people living with HIV are diagnosed late in the course of the disease [4]. In Europe, Mocroft et al. [5] showed that although late presentation for HIV care has decreased over time, it remains a significant issue in all HIV exposure groups. Over the 2000–2011 period, more than 50% of HIV-infected people were diagnosed late (defined as diagnosis with a CD4 count below 350 cells/μL or an AIDS diagnosis in the first 6 months after diagnosis) and a third presented very late (defined as diagnosis with a CD4 count below 200 cells/μL). A recent update demonstrated no overall change in the proportion of late presentation since 2010 [6].

At the population level, late diagnosis drives the existence of a hidden epidemic, with a number of HIV-infected individuals remaining unaware of their HIV status for a considerable time before diagnosis [7]. HIV-infected people who are unaware of living with HIV cannot benefit from highly effective treatment and may unwillingly contribute to the on-going transmission of HIV infection [8]. In 2016, an estimated 101,400 people were living with undiagnosed HIV in the European Union/European Economic Area (EU/EEA) and the rate of new infections was 3.6 per 100,000 population. Despite the positive trend in reduced numbers of undiagnosed HIV infections in recent years, the median time from HIV infection to diagnosis was estimated at 2.9 years in 2016 [9].

Early diagnosis with prompt link to care and treatment therefore remains a priority in the fight against the HIV epidemic [10]. It is acknowledged that general practitioners (GPs) have a pivotal role in HIV testing for early case finding [11–15]. GPs are the main entry point into the health care system for a variety of health issues, including HIV. Moreover the long-term and holistic patient-doctor relationship provided by primary care services lends itself to the provision of personalised sexual health information and repeated testing opportunities [16]. However, several studies have shown that HIV-infected individuals who are diagnosed late, often have a history of missed opportunities for an earlier diagnosis, including multiple visits to primary care services [17, 18].

To further promote HIV testing in primary care settings, to design new interventions and to increase the utility of the available research, the existing evidence should be synthesised and diffused. To that end, we conducted a mixed-methods systematic review aimed at the integration of results from both qualitative and quantitative studies on this topic [19]. The research synthesis was guided by the following research question: what are the current practices of HIV testing in primary care settings in Europe, exploring the process from targeting patients to performing the test and results communication, identifying also the barriers and facilitators.

## Methods

Mixed methods research, the paradigm that encourages the combined use of qualitative and quantitative research techniques, can be applied at the primary empirical study level as well as at the synthesis level. In such a synthesis, the data to be included in the review are findings extracted from qualitative, quantitative, and mixed methods primary level studies [20]. As the findings of various types of research dedicated to the present topic can be viewed as confirming and extending each other, an integrative mixed research synthesis as proposed by Sandelowski et al. [19] has been used. This mixed-methods systematic review was performed in accordance with the Preferred Reporting Items for Systematic Reviews and Meta-analysis (PRISMA) guidelines [21] (see Additional file 1).

### Search strategy

In May 2017, the databases PubMed, Scopus and Embase were searched for studies on HIV testing in general practices in Europe. We applied a free text strategy and MeSH terms to systematically scan the databases. In order to ensure a comprehensive search, we employed a combination of broad search terms at the level of the title and abstract. The search terms “HIV testing” and “HIV screening” were combined using the Boolean and proximity operator OR. Also the terms “general practitioners”, “general practice”, “primary care”, “family practice”, “family practitioners” were combined with OR. The terms of both areas were merged with AND. The search strategy that we used in PubMed is given in Additional file 2; this search strategy was adapted accordingly for the Scopus and the Embase databases.

### Inclusion and exclusion criteria

To be eligible, articles needed to be published in English between 2006 and 2017 in a peer reviewed journal, and report on empirical studies regarding HIV testing in general practice settings in Europe, taking into consideration at least one of the following aspects of HIV testing: sexual history taking, targeting patients, test proposal, performance, barriers and facilitators. We looked for peer-reviewed articles published from 2006 onwards as 2006 was the year in which guidelines for provider-initiated HIV testing were first released [22]. Conference proceedings, editorials and opinion papers were excluded from the review. Articles reporting on HIV testing in multiple settings without referring specifically to results for general practices, in community based settings (outreach) and on the effectiveness of indicator-disease-based testing if not specifically referring to HIV testing attitudes and practices of GPs, were also excluded.

General practice settings were defined as places where first-contact, non-specialised, long-term person-focused and comprehensive care for most health problems is provided by a physician in a sustained partnership with patients and in the context of family and community [23].

### Study selection

The search for studies was done separately in each database. The study selection followed a two-step process: review of titles and keywords followed by an abstract review. Two of the authors independently screened all identified study titles. After removal of the duplicates, results from this screening were compared and those not deemed relevant were disregarded. Abstracts from selected studies were then assessed by both authors using the above eligibility criteria. The selected abstracts from the three databases were assembled and full text reports of selected studies were subsequently analysed and checked again for eligibility by both authors. The reference lists of the papers retrieved from the search in the databases were reviewed for additional relevant references using the same eligibility criteria.

### Quality appraisal

One single checklist, adapted from Fakoya et al. [24, 25], was devised to assess the quality of both the selected quantitative and qualitative studies. Studies were given a quality score which incorporated a number of factors drawn from the PRISMA [21] and NICE guidelines [26] including the definition of a research question, description of the results, internal and external validity (see Additional file 3). For the quality appraisal of the qualitative studies we adapted the criteria from the checklist developed by Fakoya et al. [25] based on generally accepted criteria of scientific rigor in qualitative research [27]. Since qualitative research follows different research paradigms than quantitative research, we developed parallel items reflecting methodological soundness in qualitative research. To assess external validity, we used the concept of transferability, i.e. results can be applied to other similar populations and settings, which requires a sufficient description of the context in which the research was conducted. To assess the quality of outcome measures, we used the quality criteria of confirmability, i.e. research findings are supported by internally coherent data, including a transparent and systematic approach to data analysis such as triangulation, multiple coders, reflexivity, inclusion of discrepant results. Studies were rated within the paradigm of their study type and were graded as having an overall quality score of ‘high’, ‘moderate’, or ‘low’. The quality assessment was carried out by two of the authors. Studies that received a ‘low’ score from both authors were excluded from the review.

### Data synthesis

We performed a mixed research synthesis making use of an integrated design [9], in which studies are grouped for synthesis not by methods (i.e., qualitative and quantitative), but rather by findings perceived as addressing the same aspects of the study subject. The analytic emphasis is on transforming findings to combine them.

In this view, findings were categorised based on different aspects of the HIV testing practice namely trends of HIV testing, targeting of patients, test performance, as well as barriers and facilitators to HIV testing.

## Results

**Fig. 1:**
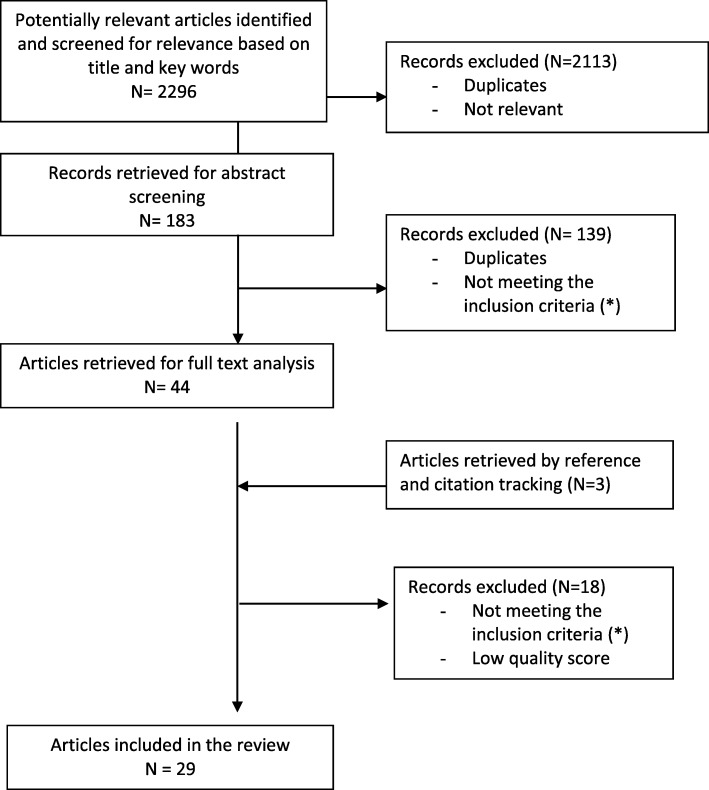
Flow diagram of the article selection process. (*) Conference proceedings, editorials, opinion papers; studies conducted outside Europe; published in other languages than English; published in journals without peer-review; published before 2006; reporting on HIV testing in other settings; reporting on HIV testing in multiple settings without singling out results for general practices; reporting on HIV testing provided by general practitioners in community based settings (outreach); reporting on the effectiveness of indicator-disease-based testing if not referring specifically to HIV testing attitudes and practices of GPs

Using the predefined search terms, 2296 potential manuscripts were identified in the three databases together (see for more information Fig. 1). After a first removal of duplicates, initial review for relevance based on title and availability of abstract, 183 abstracts remained to be screened for eligibility. After screening these abstracts, 110 articles were selected of which 44 articles were retrieved for full text analysis using the inclusion criteria. Three additional papers were found by checking the citations in the selected articles. In total 30 articles met the eligibility criteria. Based on the quality assessment, 29 studies were rated to have a high or moderate score. One mixed method study was rated with a low score as it did not provide sufficient information concerning the criteria of confirmability and transferability.

Twenty three quantitative studies were included, five qualitative studies and one had both a quantitative and qualitative approach. The studies were conducted in the United Kingdom (UK) (*n* = 9), France (*n* = 6), the Netherlands (n = 6), Belgium (*n* = 5) and Spain (*n* = 3). A few studies were done in multiple settings, including general practice. In these cases, only the results regarding HIV testing in general practice settings were singled out. Some studies dealt with one aspect of the HIV testing practice whilst others examined various topics.

### Trends in HIV testing

Little is known about trends in HIV testing in general practice. A retrospective cohort study between 1995 and 2005 in the UK showed that HIV testing rates in primary care increased slowly but remained low at 71.3 and 61.1 tests per 100,000 persons year for males and females respectively [28]. A Dutch time-trend analysis comprising a 22-years period revealed a significant increase in HIV-related consultations from 7 per 10,000 registered patients in 1988 to 13 per 10,000 patients in 2009. An analysis of the number of HIV tests demonstrated an increase from 53% of patients being tested in 1988 to 88% in 2009. There was, however, a wide variation between general practices, with HIV tests being more often requested and performed in urban areas compared with rural areas [29]. A UK study making use of chlamydia and HIV testing data from general practices indicated substantial differences in the intensity of testing. Practices that tested more tended to have younger GPs who were more likely to have had education about sexually transmitted infections (STI) and HIV in their vocational training. As a consequence, they felt more comfortable discussing sexual health issues [30]. (See Table 1 for more information).

**Table 1:** Studies included in the review that report on trends in HIV testing

Author, year	Country	Study design	Study population	Main findings
Donker, 2013 [29]	Netherlands	Retrospective cohort study within the Dutch sentinel general practice network, 1988–2009	56 GPs^a^ in 42 general practices	Increase from 53% of patients consulting for HIV and being tested in 1988 to 88% in 2009.
Evans, 2009 [28]	UK	Retrospective cohort study of all general practices contributing data to the UK General Practice Research Database, 1995–2005	13.8 million person years of observation for males and 13.9 million person years for females	11-fold increase in male testing and 19-fold increase in non-pregnant female testing between 1995 and 2005. HIV testing rates in 2005: 71.3 and 61.2 tests per 100,000 person years for males and females respectively.
Sadler, 2010 [30]	UK	Cross-sectional study using laboratory data from primary care settings in Brent and Avon, 2003–2006	207 general practices	Mean yearly HIV testing rate: 0.6 per 1000 patients aged 15–44 years in Brent and 10.3 in Avon. GPs in practices that tested for HIV were younger: mean age 50.1 years versus 54.5 years.

^a^*GPs* general practitioners

### Targeting patients

Targeting patients for HIV testing requires awareness about their HIV risk. A Belgian qualitative study showed that GPs were not inclined to routinely collect and record sexual health information from their patients. Sexual behaviour was hardly ever discussed except in cases of genital complaint. Instead, assumptions about HIV risk were based on certain patient characteristics such as country of origin, past STI episodes and sexual orientation [31]. A cross-sectional survey among newly diagnosed HIV patients (*N* = 111) in Amsterdam (Netherlands) revealed that sexual orientation was registered in the patients’ record in 34% of the men having sex with men (MSM) cases. Information about the patient origins in HIV endemic countries was collected for 56% of the patients in the migrant group [32]. In the Belgian network of Sentinel General Practices, sexual history reporting was incomplete for 55% of the STI episodes recorded between 2013 and 2014, particularly in terms of number of partners and condom use [11].

With a view to better targeting patients for an HIV test in healthcare settings, multiple guidelines and recommendations have been developed. For example, the British HIV Association, the British Association of Sexual Health and HIV and the British Infection Society introduced joint guidelines in 2008 with specific criteria applicable to general practice [33]. A questionnaire survey conducted in 2012 among GPs (*N* = 80) from both low and high HIV prevalence areas in the UK showed, however, that about half of them were unaware of these guidelines; another third was aware of the existence of the guidelines but had never read them [34]. A similar study among GPs in Paris, France (*N* = 407) revealed that only 45% of them were aware of the national recommendations for HIV screening in general practice [35].

Different approaches to target patients for testing were examined. A few studies addressed the provision of routine HIV testing to all patients. It was perceived as a way to reduce HIV-related stigma [36]. Two studies in France (*N* = 352; *N* = 78) and another in the UK (*N* = 144) reported that a majority of GPs were favourable to implement routine testing [37–39]. Nevertheless, a study among GPs in the Pays de Loire (France) (*N* = 871) indicated that routine screening of all patients was not a standard practice [40]. Instead HIV testing was offered to populations at high risk for HIV acquisition, including people who inject drugs, sex workers, MSM and people coming from high HIV endemic countries [37, 40].

The guidance released by the HIV in Europe Initiative in 2012 recommends the provision of HIV testing for patients presenting with indicator diseases, defined as co-morbid diseases afflicting HIV infected persons disproportionally [41]. GPs in the Netherlands (*N* = 81) considered that the list of HIV indicator diseases as too long and not applicable in its current form in primary care [42]. In a survey among GPs in the UK (*N* = 80), the majority indicated that it would be feasible to routinely offer HIV testing to patients with the following HIV indicator diseases: STIs and multidermatomal or recurrent herpes zoster infection. Marked variation was seen, however, in attitudes towards testing in patients presenting with other clinical HIV indicator diseases [34]. Regarding the implementation of indicator-condition-guided HIV testing, two Spanish studies revealed that testing based on selected indicator conditions commonly seen in primary care settings was a feasible and efficient strategy to improve diagnosis of HIV infection [43, 44]. HIV testing, however, was only performed in a minority of cases, with the highest testing rates when the indicator condition episode included an STI [43].

Offering an HIV test during STI consultations was examined in more detail in a few observational studies. A first study implemented between 2008 and 2011 in the Dutch sentinel GPs network showed that HIV tests were not carried out for 64% of the STI-related consultations involving patients at higher risk for HIV, and in a more recent study in the same setting for 34% [45, 46]. In particular, MSM and persons originating from HIV-endemic countries were frequently not tested. The main reasons for not testing for HIV were that the GP considered that the individual risk for HIV infection was low or that the patient did not want to be tested or to discuss the topic of HIV during the STI consultation [45]. In the Belgian network of Sentinel General Practices, 23% of the STI patients never had an HIV test prior to the present STI [11]. A search in the electronic general practice database from 747 GPs in Rotterdam (Netherlands) revealed that for 32% of persons diagnosed with syphilis, no HIV test was reported. For gonorrhoea this was 45%, for chlamydia 54% and for hepatitis B 61% [47] (see Table 2 for more information).

**Table 2:** Studies included in the review that report on targeting patients

Author, year	Country	Study design	Study population	Main findings
Agusti, 2016 [43]	Spain	Retrospective observational study making use of data from a population-based public health database, 2010–2012	99,426 patients diagnosed with an IC^a^ in primary care (*N* = 102,647 diagnosed ICs)	An HIV test was performed within 4 months in 18,515 of the episodes in which an IC was diagnosed (18,5%).
Boffin, 2017 [11]	Belgium	Retrospective observational study making use of data from the Belgian Network of Sentinel General Practices, 2013–2014	306 new STI^b^ episodes from 298 patients, reported by 83 of 140 sentinel practices [no STIs were reported by 57 practices].	For 54.6% of all STI episodes an incomplete sexual history was reported: the highest proportion of missing values was found for the number of sex partners in the past 6 months (37.6%) and condom use (25.2%). One in three STI patients (33.1%) had never been tested for HIV. Excluding those episodes for which an HIV test was planned, 23.3% never had a test (planned).
Fraisse, 2015 [37]	France	Cross-sectional questionnaire survey, 2013	78 GPs^c^ working in a 150,000-population district in the south of France	For high risk populations, including PWID,^d^ sex workers, MSM^e^ and people coming from high endemic countries, 61% of the GPs proposed HIV testing once a year.
Hall, 2015 [40]	France	Cross-sectional questionnaire survey among family physicians in the Pays de la Loire, 2011–2012	871 GPs	Routine screening of all patients was not a standard practice. 72.5% of GPs offered an HIV test to pregnant women; 70.2% to patient engaging in unsafe sex; 38.6% to MSM; 19.9% to patients presenting with symptoms of HIV and 12.5% to patients of African origin.
Hindocha, 2013 [34]	UK	Cross-sectional questionnaire survey, 2012	80 GPs from areas of high and low HIV prevalence	44% of GPs were unaware of national HIV testing recommendations. 88% would routinely test STI patients; 4% would routinely test all patients with one of the 10 ICs considered most prevalent in primary care.
Joore, 2017 [32]	Netherlands	Cross-sectional questionnaire survey among newly diagnosed HIV infected patients presenting at 2 HIV outpatient clinics in Amsterdam, 2014–2016	111 newly diagnosed HIV patients	Sexual orientation was registered in the patients’ records in 34% of the MSM cases. Information about the patient origins in HIV endemic countries was registered for 56% of the patients from the migrant group.
Joore, 2016 [42]	Netherlands	Qualitative study with FGDs and in depth-interviews, 2014	6 FGDs^f^ including 81 GPs and in- depth interviews with 9 key-informants	The list of IC is too long and therefore not applicable in its current form in primary care.
Joore, 2016 [45]	Netherlands	Retrospective observational study making use of a consultation-based data set from the Sentinel Practices of the Primary Care Database, 2008–2013, combined with a questionnaire survey among GPs	907 STI-related consultations in high risk groups	No HIV test in 34% of the STI-related consultations in patients from high risk groups. Main reasons for not testing for HIV: patient not at risk; time of risk exposure was too recent; patient refused to be tested.
Joore, 2016 [47]	Netherlands	Cross-sectional search in an electronic general practice database containing records from 747 GPS in Rotterdam, making use of a case-control design, 2009–2013	224 HIV cases which were matched with 2193 controls	In 32.1% of persons diagnosed with syphilis, no HIV test was reported in the medical records. For gonorrhea this was 44.7%, for hepatitis B 61.5% and for chlamydia 54.2%.
Menacho, 2013 [44]	Spain	Interventional study in 4 primary care centers in Barcelona, 2009–2011	775 patients included in the group with ICs and 6604 patients in the group without ICs	Testing based on selected ICs commonly seen in general practice was feasible and more efficient than non-targeted HIV testing.
Poirier, 2015 [38]	France	Multi-center observational and interventional study offering rapid HIV testing, 2012–2013	352 GPs participating in the questionnaire survey and 23 GPs volunteering to use rapid testing	71% in favour of global screening of the population from 15 to 70 years of age, without any risk factor.
Rayment, 2012 [39]	UK	Multi-center cross-sectional questionnaire survey combined with and interventional study offering HIV testing, 2009–2010	144 primary care staff, 1320 primary care patients	75% of primary care staff felt comfortable to provide HIV testing to all patients.
Rochetti, 2015 [35]	France	Cross-sectional questionnaire survey, 2012	407 GPs in Paris	45% of GPs were aware of national HIV testing recommendations; only 8% had been trained to apply these recommendations. Diagnosis of a STI was systematically considered as a case requiring HIV screening for 80% of the GPs.
Thornton, 2012 [36]	UK	Qualitative study with FGDs embedded within an interventional study offering routine testing in non-traditional settings including primary care, 2009–2010	6 FGDs in the pre-testing phase including 10 GPs; 7 FGDs in the post-testing phase including 8 GPs	Routine testing was perceived as a useful tool to reduce HIV-related stigma.
Trienekens, 2013 [46]	Netherlands	Prospective observational patient survey within the representative Dutch sentinel GP network, 2008–2011	43 general practices; 2111 new episodes concerning STI/HIV issues	No HIV tests were carried out for 64% of the STI-related consultations involving patients at higher risk for HIV.
Vos, 2016 [31]	Belgium	Qualitative study making use of in-depth interviews, 2011	13 GPs in urban areas in Flanders	No inclination to routinely collect and register sexual health information. Assumptions on HIV risk based on country of origin, past STI episodes and sexual orientation.

^a^*IC* Indicator condition

^b^*STI* sexually transmitted infection

^c^*GPs* general practitioners

^d^*PWID* people who inject drugs

^e^*MSM* men having sex with men

^f^*FGDs* focus group discussions

### How the HIV test is performed

Offering an HIV test without a patient’s specific request, an immediate diagnostic need, or outside of ‘a window of opportunity’ such as a blood test for other clinical indications or a sexual or reproductive health consultation caused feelings of discomfort among GPs, who expressed concern with regard to the patient’s potential reaction [42, 48, 49]. Therefore, HIV testing practices were generally limited to the above-mentioned situations [29, 32, 35–37, 40, 49]. However, this observation is not applicable to all settings. In Paris, for example, GPs (*N* = 407) were pro-actively involved in HIV screening by prescribing a substantial number of tests without waiting for the patient’s request [35].

A qualitative study in Flanders (Belgium) assessing HIV testing conditions showed that informed consent was often not specifically asked for. GPs believed that patients were sufficiently informed to accept or refuse the HIV test. Negative test results were communicated either by phone or during a follow up consultation. Post-test counselling was generally not provided when test results were negative. Positive test results were communicated personally, with focussed messages to alleviate the first shock after diagnosis, to give information about effective treatment and to refer to HIV care. However, some GPs did not offer any post-test counselling; they simply referred patients to a specialised HIV clinic [49]. In the French study among GPs (*N* = 78), 69% reported that they had never communicated a diagnosis of HIV infection to their patients [37].

Regarding the type of HIV tests used, studies in both Spain (*N* = 1308) and France (N = 78) found that few GPs were aware of rapid testing for HIV, even among those who provided other rapid diagnostic tests [37, 50]. Nevertheless, the use of rapid tests, both on oral fluid and finger prick blood, was received favourably. It was perceived as quick, highly acceptable to patients and as reducing the risk of patients not coming back for their test results [36–38, 50, 51]. However, many GPs did not know how to use them [38, 50]. Potential test interpretation errors, the complexity of quality control and the significantly longer consultation time were considered as obstacles to the use of rapid tests in daily practice [37, 38, 50, 51]. The immediacy of the screening result was viewed as too intrusive, both to patients and GPs [38]. Some GPs were not interested in using rapid testing due to the difficulty of screening for other STIs simultaneously [37, 50]. A longer window period compared to the latest generation laboratory test was also reported as an obstacle to the use of rapid tests [51] (see Table 3 for more information).

**Table 3:** Studies included in the review that report on how the HIV test is performed

Author, year	Country	Study design	Study population	Main findings
Agusti, 2013 [50]	Spain	Cross-sectional questionnaire survey, 2012	1308 GPs^a^ from the two largest Spanish scientific medical societies for family and community medicine	70% not knowing how to use a rapid HIV test; 80% willing to use it.
Donker, 2013 [29]	Netherlands	Retrospective cohort study within the Dutch sentinel general practice network, 1988–2009	56 GPs in 42 general practices	For the period 1988–2009, 77 to 93% of HIV tests were based on the patient’s request.
Fraisse, 2015 [37]	France	Cross-sectional questionnaire survey, 2013	78 GPs working in a 150,000-population district in the south of France	Main reasons for HIV testing were patient request (91%) and risk of HIV infection (62%). 69% of GPs never communicated an HIV diagnosis. 33% of GPs were informed about rapid HIV testing; 85% agreed with training on rapid HIV testing.
Gauthier, 2012 [51]	France	Prospective interventional study offering rapid testing in primary care, 2010	62 GPs and 383 primary care patients, covering six French regions + 72 GPs participating in the evaluation post intervention	60% of GPs were willing to use rapid tests for HIV.
Hall, 2015 [40]	France	Cross-sectional questionnaire survey among family physicians in the Pays de la Loire, 2011–2012	871 GPs	HIV testing practices were mostly risk-based driven or as part of a diagnostic procedure.
Joore, 2016 [42]	Netherlands	Qualitative study with FGDs and in depth-interviews, 2014	6 FGDs^b^ including 81 GPs and in- depth interviews with 9 key-informants	GPs tend to cling to risk-based HIV testing.
Joore, 2017 [32]	Netherlands	Cross-sectional questionnaire survey among newly diagnosed HIV infected patients presenting at 2 HIV outpatient clinics in Amsterdam, 2014–2016	111 newly diagnosed HIV patients	In the 5 years prior to HIV diagnosis, 82.9% of the 111 patients had one or more consultations with their GP; 34.8% had one or more HIV tests performed in general practice during this period. In more than 50% of the cases the positive test was done on the request of the patient.
Loos, 2014 [48]	Belgium	Qualitative evaluation making use of FGDs and in-depth interviews, 2011–2012	65 GPs in Flanders implementing a tool to proactively offer HIV testing to Sub-Saharan African migrants	Suggesting an HIV test without a patient’s request needs a window of opportunity such as a blood test for other medical reasons.
Manirankunda, 2012 [49]	Belgium	Qualitative study making use of in-depth interviews, 2007–2008	20 GPs in the cities of Ghent and Antwerp	HIV testing was mostly patient-initiated. No explicit informed consent was asked; no pre-test counselling; personal communication of positive test results.
Poirier, 2015 [38]	France	Multi-center observational and interventional study offering rapid HIV testing, 2012–2013	352 GPs participating in the questionnaire survey and 23 GPs volunteering to use rapid testing	77% of GPs was in favour of using rapid testing.
Rochetti, 2015 [35]	France	Cross-sectional questionnaire survey, 2012	407 GPs in Paris	74% of GPs had prescribed up to 10 HIV tests in the previous month; 47% had prescribed the latest HIV tests without waiting for the patient’s request.
Thornton, 2012 [36]	UK	Qualitative study with FGDs embedded within an interventional study offering routine testing in non-traditional settings including primary care, 2009–2010	6 FGDs in the pre-testing phase including 10 GPs; 7 FGDs in the post-testing phase including 8 GPs	Before the intervention, HIV testing practices were mostly risk-based driven or as part of a diagnostic procedure. The use of rapid tests during the intervention phase was received favourably.

^a^*GPs* general practitioners

^b^*FGDs* focus group discussions

### Barriers and facilitators to HIV testing

Concerns about the practical aspects of delivering HIV testing services emerged as an important issue. Lack of time was cited as an important operational barrier to conducting (rapid) HIV testing [36–38, 42, 48–52]. There was concern that offering an HIV test during a consultation for an apparently unrelated medical complaint might disturb the consultation process [36, 49]. While some took a pragmatic approach and understood that pre-test discussion could be brief and focussed, others believed that this was a complex and time-consuming process [36, 48–50].

When it came to testing migrants, language barriers and lack of culture-sensitive sexual counselling skills were reported [49, 50]. If HIV diagnosed patients cannot have access to medical follow-up and treatment, mainly in cases of an illegal situation or forced return to their country, this appeared to be a barrier for providers who may not be willing to make people face hopeless situations [49].

Finally, GPs expressed concerns about result management, in particular about communicating test results and a potential diagnosis of HIV infection [36, 37, 49, 51]. In light of these concerns, some GPs suggested that the delivery of test results was best conducted by specialised staff, such as sexual health advisors [36]. However, some others felt encouraged to increase the offer of HIV testing when new diagnoses had been made as a result of their intervention and when these individuals had been successfully linked to HIV care [36, 49].

A study in the UK indicated a need for additional training to include HIV testing as a routine part of patient care [39]. This was also the case in two Belgian studies regarding provider-initiated HIV testing targeting sub-Saharan African migrants as a key-population at risk [48, 49]. GPs identified particular training needs regarding HIV epidemiology and prevention, the treatment benefits of early diagnosis and HIV management immediately after diagnosis. Some GPs requested a supportive and user-friendly tool to proactively offer HIV testing to populations at increased risk. It was felt that such a tool could support the patient-provider communication mitigating feelings of discomfort when offering and discussing the test.

A number of studies suggested that the provision of a specific training, practical tool or promotion programme had a positive effect on the testing performance of GPs [48, 53–57]. Based on the data from an observational single-centre cohort study in a UK area of high HIV prevalence, Mahendran et al. [54] suggested that the continuous supply of initiatives that support testing in primary care, such as the designation of an HIV testing advisor, simplification of the testing process and training on HIV indicator diseases, could improve the recognition and diagnosis of HIV. The study showed that there was a significant increase in the proportion of new diagnoses made within primary care from 2.7% in 2000 to 21.2% in 2012 (*p* < 0.001). The rate of late diagnosis decreased from 89.5 to 32.9% (*p* < 0.001) and the diagnosis of recent infections increased from 15 to 40% (*p* < 0.001). In France, the launch in 2009 of a national guideline for HIV screening of the general population aged between 15 and 70 years, had a positive impact on the testing rates. For patients who saw their GP regularly, the intervention led to a 3.3% increase (95% CI: 2.8–3.8) in HIV testing in 2010, an 8.7% increase (95% CI: 7.4–10.1) by 2011, and a 20.4% increase (95% CI: 17.0–23.8) by 2013 [56].

In a high prevalence London area, a training intervention among GPs and nurses on sexual health clinical skills and sexual history taking produced a substantial increase in HIV testing rates: some surgeries increased their testing rates by more than 50% and high positivity rates (16.7/1000 tests) were achieved [55]. A cluster-randomised controlled trial in general practices in London (UK) demonstrated that a multifaceted educational programme, integrating rapid HIV testing into the registration health check, led to increased rates of HIV diagnosis with 0.30 (95% CI: 0.11–0.85) per 10,000 patients per year in intervention practices versus 0.07 (95% CI: 0.02–0.20) in control practices. A high proportion of newly diagnosed patients were of Black African ethnic origin, showing successful implementation of testing in a multi-ethnic community [53] (see Table 4 for more information).

**Table 4:** Studies included in the review that report on barriers and facilitators

Author, year	Country	Study design	Study population	Main findings
Agusti, 2013 [50]	Spain	Cross-sectional questionnaire survey, 2012	1308 GPs^a^ from the two largest Spanish scientific medical societies for family and community medicine	Barriers to provide (rapid) HIV testing: lack of time; lack of training; cultural barriers.
Fraisse, 2015 [37]	France	Cross-sectional questionnaire survey, 2013	78 GPs working in a 150,000-population district in the south of France	Barriers to provide rapid HIV testing: time constraints.
Gauthier, 2012 [51]	France	Prospective interventional study offering rapid testing in primary care, 2010	62 GPs and 383 primary care patients, covering six French regions + 72 GPs participating in the evaluation post intervention	Barriers to provide rapid HIV testing: difficulties to perform the test; lack of time; window period; difficulties to screen for other STIs.
Gennotte, 2013 [52]	Belgium	Prospective interventional study offering rapid HIV testing in a Brussels area with a substantial African community, 2010–2011	10 GPs and 1087 consultation records, 217 primary care patients offered rapid HIV testing	Barriers to provide (rapid) HIV testing: lack of time; difficulties to propose the test
Joore, 2016 [42]	Netherlands	Qualitative study with FGDs^b^ and in depth-interviews, 2014	6 FGDs including 81 GPs and in- depth interviews with 9 key-informants	Barriers to provide HIV testing: difficulties in targeting the right group; lack of time; fear of stigmatizing patients.
Leber, 2015 [53]	UK	Cluster randomised controlled trial among general practices in a multi-ethnic, socioeconomically deprived inner London borough, 2010–2011. Practices were randomised to offer either opt-out rapid testing to newly registering adults or continue usual care.	20 general practices in the intervention group and 20 in the control group	HIV diagnosis rate was 0.30 [95%CI: 0.11–0.85] per 10,000 patients per year in intervention practices versus 0.07 [95%CI: 0.02–0.20] in control practices.
Loos, 2014 [48]	Belgium	Qualitative evaluation making use of focus group discussions and in-depth interviews, 2011–2012	65 GPs implementing a tool to proactively offer HIV testing to Sub-Saharan African migrants	Barriers to provide HIV testing: feelings of discomfort to offer the test, lack of counselling skills and time constraints. GPs identified training needs on the specificities of the HIV epidemic GPs requested a tool to proactively offer HIV testing to populations at increased risk improved the testing performance.
Mahendran, 2015 [54]	UK	A single-center observational cohort study in an outpatient HIV department in a secondary care UK hospital assessing the site of initial HIV diagnosis and stage of infection, 2000–2012	1359 diagnosed HIV patients	Increase in the proportion of HIV diagnoses made in primary care: from 2.7% in 2000 to 21.2% in 2012. Decrease in the proportion of late diagnoses from 89.5% in 2000 to 42% in 2012.
Manirankunda, 2012 [49]	Belgium	Qualitative study making use of in-depth interviews, 2007–2008	20 GPs in the cities of Ghent and Antwerp	Barriers to provide HIV testing: time constraints, concerns about result management, concerns about lack of access to treatment for migrants in an illegal situation.
Pilay, 2014 [55]	UK	An interventional study consisting of a training in sexual health skills in a high HIV prevalence London area, 2010–2011	51 general practice settings	Testing rates of trained and untrained practices increased from 2.29 to 6.66 and 1.54 to 1.90/1000 registered patients/year. 16.7 positive diagnoses per 1000 tests in trained practices, corresponding to a rise from 9.5 to 22 new diagnoses per year.
Poirier, 2015 [38]	France	Multi-center observational and interventional study offering rapid HIV testing, 2012–2013	352 GPs participating in the questionnaire survey and 23 GPs volunteering to use rapid testing	Barriers to provide rapid HIV testing: difficulties to include preventive screening in GP consultation; low prevalence; immediacy of test results in case of rapid testing.
Rayment, 2012 [39]	UK	Multi-center cross-sectional questionnaire survey combined with and interventional study offering HIV testing, 2009–2010	144 primary care staff, 1320 primary care patients	72% of GPs identified a need for training to include HIV testing as a routine part of patient care.
Sicsic, 2016 [56]	France	Retrospective observational study making use of data from the French National health Insurance Fund database, 2006–2013	2.176,647 person-years corresponding to 329.748 different individuals aged between 15 and 70	Annual HIV screening rates increased from 4.2% [95% CI: 4.2–4.3] in 2006 to 5.8% [95% CI: 5.7–5.9] in 2013 with a significant trend after 2010 (*p* < 0.0001). The increase was stronger for those that regularly consulted a GP: the national screening policy led to a 20.4% increase [95% CI: 17%-23.8] in 2013 compared to a 4.5% increase [95% CI: 4.4–4.5] for those who did not consult a GP regularly in 2013.
Thornton, 2012 [36]	UK	Qualitative study with FGDs embedded within an interventional study offering routine testing in non-traditional settings including primary care, 2009–2010	6 FGDs in the pre-testing phase including 10 GPs; 7 FGDs in the post-testing phase including 8 GPs	Barriers to provide HIV testing: lack of time; concerns about results management. Routine offer to HIV testing in general practice is feasible but requires training and support for staff.
Tong, 2012 [57]	UK	Prospective interventional study adding a standard comment to encourage inclusion of HIV testing to all Glandular fever screening reports, 2010–2011	871 glandular fever screening samples from 865 patients submitted from primary care	After the introduction of the standard comment, 19.6% had a concomitant HIV request as compared to 9.5% in the baseline period.

^a^*GPs* general practitioners

^b^*FGDs* focus group discussions

## Discussion

Increasing the timely uptake of HIV testing and decreasing the number of undiagnosed people is a priority area for HIV care and prevention. International and European guidelines call vigorously for diversifying and intensifying models of HIV testing delivery within both health care and community settings [58–60]. Primary care is a frontline service providing opportunities for disease prevention, health promotion and early detection of disease. On this account it is believed that GPs have a major role to play in provider-initiated HIV testing for early case finding, which in turn may have a significant impact on the epidemic. This study is the first mixed-methods systematic review on HIV testing in general practice in Europe, summarising the available evidence on current practices and the existing barriers and facilitators to HIV testing.

This review shows that there is potential for an increased role for GPs in provider-initiated HIV testing. Available literature shows that patients usually accept being offered HIV testing in primary care, particularly when part of a general check-up or sexual health consultation [61–66]. Nevertheless, we also identified several barriers for which solutions must be identified to increase provider-initiated testing in general practice and safeguard its quality. In what is to follow, we describe the main reported barriers and propose several lines of action that could be considered to overcome these barriers.

The findings of this synthesis show that GPs are often not fully aware of the specificities of the HIV epidemic and the testing recommendations in their own countries. They request better information and training on these topics. Providing information on the HIV epidemic, notably on estimates of undiagnosed HIV prevalence and time to diagnosis by key-populations and geographical area, will be fundamental to increasing awareness and testing among GPs.

During routine GP consultations, sexual behaviours are rarely discussed and GPs feel uncomfortable with proactively offering an HIV test. HIV testing practices are mostly based on a patient’s request, a reported risk or as a part of a diagnostic procedure where knowledge of HIV status is likely to have an impact on immediate clinical management. Routinely offering an HIV test for certain HIV indicator diseases, such as STIs, has been shown to be feasible and effective. However, marked variation in attitudes and practices regarding the use of the complete list of clinical HIV-indicator-diseases has been observed. Practical tools should be developed to enable GPs to identify people at high risk of HIV infection who require regular HIV testing. For example, the DENVER HIV risk score tool calculates HIV risk scores, categorising people into groups with increasing possibilities of HIV infection, which may help to prioritise HIV testing efforts [67]. The compilation of a short-list with HIV indicator diseases commonly seen in primary care could also be an interesting option. A recent study has shown that there are indeed opportunities for HIV indicator condition-guided testing in primary care [68]. As a prerequisite for using these tools, GPs should be trained to improve skills in sexual health anamnesis, intercultural competences and accurate recognition of HIV-related conditions.

The observed lack of experience in delivering test results or communicating a diagnosis of HIV infection may be a supplementary barrier to GP’s readiness to offer HIV testing. The process of HIV testing must be in accordance with the international regulatory framework on provider-initiated HIV testing, comprising the World Health Organization ‘5Cs’ principle: Consent, Confidentiality, Counselling, Correct test results and linkage to Care [59]. Importantly, pre-test counselling and informed consent procedures should be approached pragmatically without complex and time-consuming operations, while respecting confidentiality and patients’ right to refuse testing. In addition, result management, including the delivery of HIV diagnoses and collaboration with specialised HIV clinics has to be adequately supported by education and training.

GPs’ individual time constraints have shown to be a major barrier to routinely offering HIV testing. This will remain a difficult challenge as GPs are already overburdened with the cumulating number of tasks they must complete as primary health care providers. In consequence, the roll-out of HIV testing in primary care must take into account this reality. Next to pragmatic handling of testing procedures, evidence-based brief interventions adapted to primary care settings may be helpful [69]. They aim to develop effective communication methods, like motivational interviewing, to avoid extra time for counselling and testing.

Besides addressing the main barriers identified in this review, we believe that in order to improve ‘real-world’ effectiveness, testing in primary care must be an integral part of a comprehensive HIV testing strategy based on epidemiological evidence. Indeed, detailed mapping and sub-national estimates of undiagnosed HIV prevalence, of late HIV diagnosis and of HIV incidence are fundamental to identify and effectively reach-out to the most affected populations in each location. These estimates are essential to raise awareness among the public and GPs, to determine whether testing interventions increase timely HIV diagnosis and to assess their cost-effectiveness on a larger scale. A recent study produced these key estimates at a granular level in France using detailed national HIV surveillance data [70]. However, they remain to be produced at national and sub-national level in most settings.

Finally, HIV testing programs should be integrated within a balanced combination prevention framework, including biomedical, behavioural and structural interventions that address the complex interplay of underlying determinants of HIV transmission. All this requires equal access to a continuum of HIV prevention, testing and care services for all without discrimination, and implemented through a multi-sectorial and participatory approach [66, 71].

There are some limitations to our review. Studies were only included if they had been published as research articles in English-language peer-reviewed journals. Relevant data from grey literature or publications in other languages were therefore excluded from this review. Another limitation derives from the sparse literature available, making a cross-European comparison impossible. One third of the retrieved studies were performed in the UK, a limited number were conducted in alternate Western-European countries, but none originated from other European regions. This may be partially explained by the national differences in health care delivery models and screening organisation. We also acknowledge that some of the implementation and evaluation studies included in this review were operating under ideal circumstances, and the positive results obtained may not be achievable in the real life conditions of busy primary care practices.

## Conclusions

This review provides evidence of the conditions under which GPs could play an increased role in provider-initiated HIV-testing for early case finding, which is essential to improve health outcomes and to reduce transmission risks. It will be necessary to further elaborate on the identified solutions to reported barriers and to define specific testing criteria adapted to primary healthcare. This process should be conducted in collaboration with relevant stakeholders such as GPs, patient organisations and policy makers. Since it is a complex task to incorporate changes into clinical practice [72], further studies will be needed to promote and evaluate HIV testing in primary care, including its impact, effectiveness, quality and associated costs.

## Additional files

Additional file 1: Reported Items of the PRISMA checklist. (PDF 29 kb)

Additional file 2: Sample search strategy in PubMed. (PDF 22 kb)

Additional file 3: Quality assessment tool. (PDF 51 kb)

## Abbreviations

CD4: Cluster of Differentiation 4
EEA: European Economic Area
EU: European Union
GP: General Practitioner
HIV: Human Immunodeficiency Virus
MSM: Men having sex with men
STI: Sexually Transmitted Infection
