# Supplementary material for: HIV testing within general practices in Europe: a mixed-methods systematic review
Source: BMC Public Health. 2018 Oct 22;18:1191. doi: 10.1186/s12889-018-6107-0 (PMC6196459; doi:10.1186/s12889-018-6107-0)
Supplement: Supplementary file 2 — Sample search strategy in PubMed. (PDF 22 kb) [file 12889_2018_6107_MOESM2_ESM.pdf]

Additional file 2: Sample search strategy in PubMed

#1("hiv"[MeSH Terms] OR "hiv"[All Fields]) AND testing[All Fields]

#2("hiv"[MeSH Terms] OR "hiv"[All Fields]) AND ("diagnosis"[Subheading] OR "diagnosis"[All Fields] OR "screening"[All Fields] OR "mass screening"[MeSH Terms] OR ("mass"[All Fields] AND "screening"[All Fields]) OR "mass screening"[All Fields] OR "screening"[All Fields] OR "early detection of cancer"[MeSH Terms] OR ("early"[All Fields] AND "detection"[All Fields] AND "cancer"[All Fields]) OR "early detection of cancer"[All Fields])

#3"general practitioners"[MeSH Terms] OR ("general"[All Fields] AND "practitioners"[All Fields]) OR "general practitioners"[All Fields] OR ("general"[All Fields] AND "practitioner"[All Fields]) OR "general practitioner"[All Fields]

#4"general practice"[MeSH Terms] OR ("general"[All Fields] AND "practice"[All Fields]) OR "general practice"[All Fields]

#5"primary health care"[MeSH Terms] OR ("primary"[All Fields] AND "health"[All Fields] AND "care"[All Fields]) OR "primary health care"[All Fields] OR ("primary"[All Fields] AND "care"[All Fields]) OR "primary care"[All Fields]

#6 "family practice"[MeSH Terms] OR ("family"[All Fields] AND "practice"[All Fields]) OR "family practice"[All Fields]

#7 "family"[MeSH Terms] OR "family"[All Fields]) AND practitioners[All Fields]

#8 = (#1 or #2) and (#3 or #4 or #5 or #6 or #7)

A filter with the following limits was used: period 2006-2017, availability of full text and Europe.

The above search strategy was adapted accordingly for the Scopus and Embase databases.
