# Supplementary material for: HIV testing within general practices in Europe: a mixed-methods systematic review
Source: BMC Public Health. 2018 Oct 22;18:1191. doi: 10.1186/s12889-018-6107-0 (PMC6196459; doi:10.1186/s12889-018-6107-0)
Supplement: Supplementary file 3 — Quality assessment tool. (PDF 51 kb) [file 12889_2018_6107_MOESM3_ESM.pdf]

Additional file 3: Quality assessment tool

	Very well	Well	Moderate	Poor	Not informed	Not applicable
**Q1. Research question is clearly defined.**						
**Q2. Internal validity. The study has a good design, appropriate for their objectives and minimizing design bias.**						
**Q3. Results are well described, useful and accurate described.**						
**Q4.1. For quantitative studies: external validity. Results are generalizable to the population and the context which is interesting to apply them.**						
**Q4.2. For qualitative studies: transferability. Results can be applied to other similar populations and settings, which requires a sufficient description of the context in which the research was conducted.**						
**Q5.1. For quantitative studies: strength of association and statistical significance. Outcomes are precise, with narrow confidence intervals and/or low p-values.**						

**Q5.2. For qualitative studies: confirmability. The research findings are supported by internally coherent data, including a transparent and systematic approach to data analysis (e.g. triangulation, multiple coders, reflexivity, inclusion of discrepant results)**						

	High	Moderate	Low
**Average quality of the paper**			

References

- European Centre for Disease Prevention and Control. Migrant health: Sexual transmission of HIV within migrant groups in the EU/EEA and implications for effective interventions. Stockholm: ECDC; 2013.
- Fakoya I, Álvarez-del Arco D, Woode-Owusu M, Monge S, Rivero-Montesdeoca Y, Delpech V, Rice B, Noori T, Pharris A, Amato-Gauci AJ, del Amo J, Burns FM. A systematic review of post-migration acquisition of HIV among migrants from countries with generalized HIV epidemics living in Europe: implications for effectively managing HIV prevention programmes and policy. BMC Public Health. 2015 Jun 19;15:561. DOI: http://dx.doi.org/10.1186/s12889-015-1852-9
- Given, LM (2008). The Sage Encyclopedia of Qualitative Research. DOI: http://dx.doi.org/10.4135/9781412963909.n60
- Stacy M. Carter, Jan E. Ritchie, Peter Sainsbury. Doing good qualitative research in public health: not as easy as it looks. New South Wales Public Health Bulletin 20(8) 105–111 DOI: http://dx.doi.org/10.1071/NB09018 Published online: 7 September 2009
- Methods for the Development of NICE Public Health Guidance [Internet]. Editors National Institute for Health and Care Excellence. Source London: National Institute for Health and Care Excellence (NICE); 2012 Sep. Process and Methods Guides No. 4.
